# Prevalence of *Helicobacter pylori* infection in patients with chronic hepatitis C

**DOI:** 10.1186/s43141-021-00293-1

**Published:** 2022-01-26

**Authors:** Abdelfattah M. Attallah, Mohamed S. Albannan, Mohamed F. Ghaly, Sally E. Sallam, Mahmoud M. Amer, Attia A. Attia

**Affiliations:** 1 Biotechnology Research Center, 23 July St., Industrial Zone, New Damietta, 34517 Egypt; 2 Faculty of Science, Zagazig University, Zagazig City, Egypt; 3 Faculty of Science, Benha University, Benha City, Egypt

**Keywords:** *Helicobacter pylori*, Co-infection, Liver, Fibrosis, Cirrhosis

## Abstract

**Background:**

Association between *Helicobacter pylori* (*H. pylori*) and chronic hepatitis C (CHC) still remains controversial. This work is concerned with assessing the potential role of *H. pylori* in the progression of hepatitis C virus (HCV)-related chronic liver disease.

**Results:**

A total of 449 individuals constituted this study (200 individuals were used to validate the assay while 249 individuals were used to assess the correlation between *H. pylori* infection and CHC). *H. pylori* antigen was quantified in serum samples using ELISA. As a consequence, our findings showed that *H. pylori* positivity was increased significantly (*P* = 0.021) with liver fibrosis progression as it was found in 44.45% of fibrotic patients and 71.88% of cirrhotic patients. We demonstrated that patients with F4 were accompanied by a significant (*P* < 0.05) increase in the concentration of *H. pylori* antigen displaying 16.52-fold and 1.34-fold increase in its level over F0 and F1-F3, respectively. Patients co-infected with *H. pylori* and HCV are 3.19 times (219%) more likely to experience cirrhosis than those who are mono-infected with HCV. This suggests that the risk for developing F4 was found to increase upon *H. pylori* co-infection when compared to CHC mono-infected patients.

**Conclusion:**

The elevated levels of *H. pylori-antigen* in HCV/*H. pylori* co-infection suggest increased susceptibility of co-infected patients for promoting hepatic fibrosis progression.

## Background

The discovery of *Helicobacter pyl*ori (*H. pylori*) has without doubt been one of the most remarkable achievements in medical research in the past three decades. *H. pylori* infects 50% of the world population. Its prevalence varies widely in different parts of the world with higher rates in developing countries. In most instances, it is acquired during childhood, and is often associated with low socio-economic class [1, 2]. It produces chronic gastritis by provoking local inflammatory response in the epithelium through release of a range of cytokine [3]. The World Health Organization classifies the bacterium as a class I carcinogen and a close association between infection and 70% of gastric cancer cases worldwide has been reported [1, 2]. Additionally, liver is likely a probable organ to be affected by *H. pylori* activity [4–6]. In this case, clinicians would need a rapid and reproducible, noninvasive assay for the screening of patients for the presence of this pathogen and if a patient indeed tested positive for *H. pylori* for monitoring the success of eradication therapy [7]. Unfortunately, *H. pylori* is fastidious and difficult to cultivate [8]. Therefore, detecting serum *H. pylori* antigen offers an alternative noninvasive diagnostic test. In this respect, we previously developed a sensitive and specific noninvasive immunoassay based on the detection of *H. pylori* circulating antigen in sera from *H. pylori*-infected individuals with high degree of accuracy [9]. On the other hand, increasing evidence suggests that *H. pylori* may be a risk factor for developing cirrhosis in patients with chronic hepatitis C (CHC) [10, 11].

Herein, we are concerned with achieving two objectives. The first one is concerned with identification and quantitation of *H. pylori* antigen in CHC patients and validate whether the predictive criteria identified in the previous study were able to reproduce their predictive ability in a subsequent different, but related, group of patients. The second one is dedicated to assessing the potential role of *H. pylori* infection in the progression of hepatitis C virus (HCV)-related chronic liver disease.

## Methods

### Samples

A total of 449 consecutive Egyptian individuals composed of three different groups constituted the present study. Informed consents were obtained from all participants and they were fully informed concerning the diagnostic procedures involved and disease nature. The study protocol was approved by the Ethics Committee of the Mansoura University hospitals, Mansoura University, Egypt. The consent to participate was obtained verbally and approved by Ethics Committee. The first group included serum samples of 100 *H. pylori*-infected individuals collected from the Gastro-Enterology and Surgery Center, Mansoura University, Mansoura, Egypt. They were diagnosed based on culture as a gold standard.

The second group included serum samples of another 249 chronic hepatitis C patients [95 with F0, 90 with F1‑F3, and 64 with F4] collected from the Tropical Medicine Department, Mansoura University hospitals, Mansoura, Egypt. These patients were tested positive for the presence of HCV-ribonucleic acid (RNA) using quantitative polymerase chain reaction assay (COBAS Ampliprep/COBAS TaqMan, Roche Diagnostics, Pleasanton, USA). Histopathological classification for liver fibrosis and cirrhosis was performed according to the METAVIR score [12]. Liver fibrosis was defined as a METAVIR score of ≤ 3 (F1‑F3) whereas cirrhosis was defined as a METAVIR score of 4 (F4). The third group included serum samples of another 100 healthy volunteers used as a control group. Overall, the first and the third groups were used to validate the assay while the second group was dedicated to assessing the correlation between *H. pylori* infection and chronic hepatitis C.

### Laboratory tests

All serum samples constituted this study were subjected to laboratory investigations including liver function tests [alanine aminotransferase (ALT), aspartate aminotransferase (AST), total bilirubin, albumin, and alkaline phosphatase (ALP)] measured on an automated biochemistry analyzer (A15, Biosystem, Spain). Alpha fetoprotein (AFP) level was performed by chemiluminescence, with Immulite AFP (1000) kit (Diagnostic Products Corporation; Los Angeles, CA, USA).

### Detection of 58-kDa H. pylori antigen using ELISA

First of all, the target 58-kDa *H. pylori* antigen was previously identified at 58-kDa molecular weight based on sodium dodecyl sulfate-polyacrylamide gel electrophoresis followed by Western blot as published by Attallah et al. [9]*.* In this work, the quantification of 58-kDa *H. pylori* antigen was performed based on ELISA technique using its mono-specific antibodies (ABC Diagnostics, New Damietta, Egypt) as according to Attallah et al. [9]. Color intensity was proportional to the amount of bound conjugate and therefore is a function of the concentration of *H. pylori* antigen present in the serum sample.

### Statistical analysis

All statistical calculations were done by the SPSS software v.15.0 (SPSS Inc., Chicago, IL) and GraphPad Prism package v.5.0 (GraphPad Software, San Diego, CA). Continuous variables were expressed as mean ± standard error of mean. One-way analysis of variance (ANOVA) and Tukey’s post hoc test were used to compare continuous variables while chi-square test was used to compare proportions. All tests were two-tailed and statistical significance assessed at the 0.05 level. Area under the curve (AUC) was used to assess the diagnostic value of 58-kDa *H. pylori* antigen to distinguish between patients infected with *H. pylori* and non-infected individuals. An AUC equal to 1.0 is characteristic of an ideal test, whereas 0.5 indicates a test without diagnostic value. The nearer a curve shifts to the top left-hand corner of the graph, the more useful the marker is for the diagnosis. Results obtained by the *H. pylori* culture test as a gold standard; common indicators of 58-kDa *H. pylori* antigen accuracy (sensitivity, specificity, efficiency, and odds ratio) were derived from such a 2 × 2 contingency table. Odds ratio (with 95% confidence intervals) was calculated to estimate the risk of a target disorder from subjects without it.

## Results

**Fig. 1:**
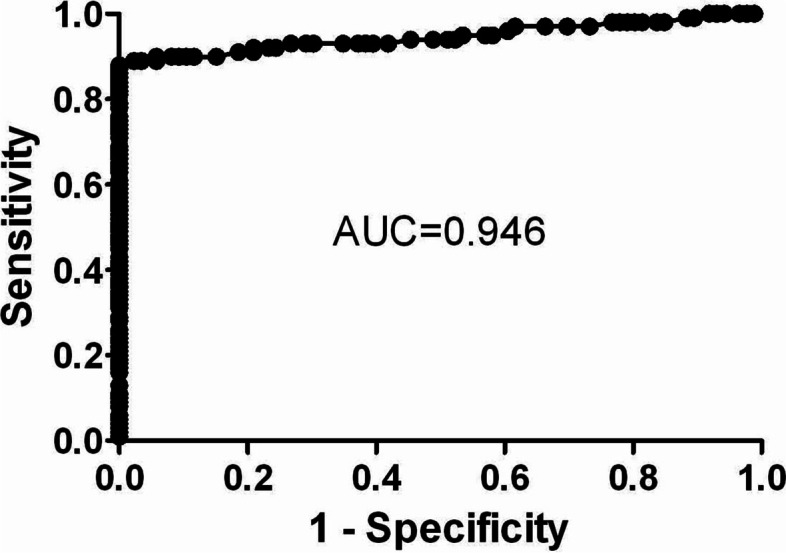
Diagnostic accuracy of 58-kDa *Helicobacter Pylori* (*H. pylori*) antigen for the diagnosis of *H. pylori* infection

As formerly mentioned, the first objective of this work is to validate whether the predictive criteria identified in the previous study [9] were able to reproduce their predictive ability in a subsequent different, but related, group of patients based on ELISA analysis. In order to estimate the diagnostic accuracy of 58-kDa *H. pylori* antigen, ROC curve was used as depicted in Fig. 1. As a result, the 58-kDa *H. pylori* antigen enabled the correct identification of *H. pylori* patients showing an AUC of 0.946 with 90% sensitivity, 90% specificity, and 90% efficiency.

**Fig. 2:**
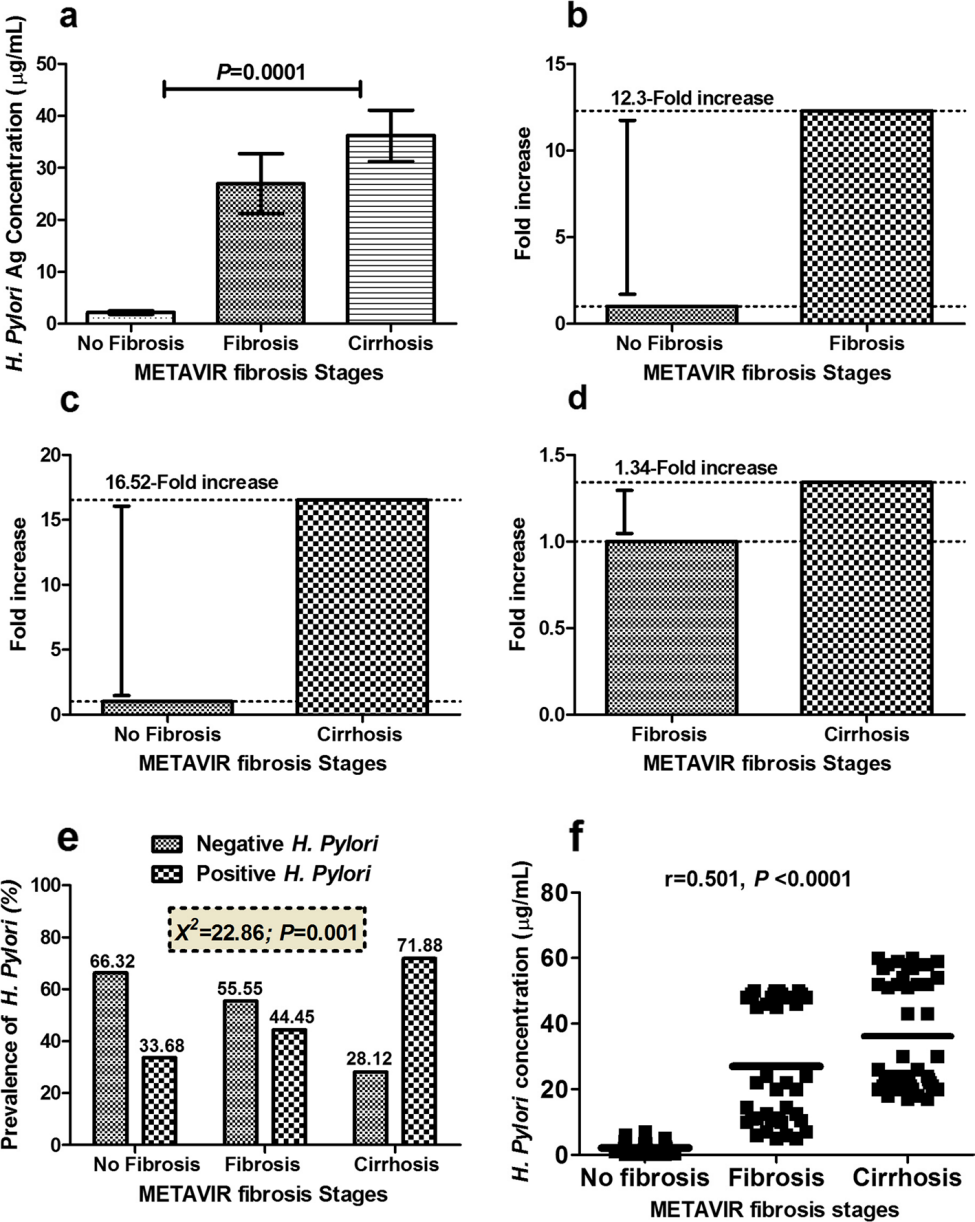
Quantification of *H. pylori* antigen in addition to its observed fold changes in serum of chronic hepatitis C patients (**a**). The levels of 58-kDa *H. pylori* antigen among different METAVIR fibrosis stages (**b**, **c**, **d**). The distribution of observed fold changes for 58-kDa *H. pylori* antigen among different METAVIR fibrosis stages (**e**). Prevalence of 58-kDa *H. pylori* antigen among patients with liver fibrosis and cirrhosis showing an increase in its detection rate with the progression of liver pathology (**f**). Scatter plot showing the distribution of 58-kDa *H. pylori* antigen with the progression of liver fibrosis. *P* > 0.05 is considered non-significant, *P* < 0.05 is considered significant, and *P* < 0.0001 is considered extremely significant

The levels of *H. pylori* antigen were then quantified in patients’ sera and the results are depicted in Fig. 2a. As a consequence, our findings demonstrated that patients who had hepatic cirrhosis were accompanied by a significant (*P* < 0.05) increase in the concentration of *H. pylori* antigen when compared to those with F0 and F1‑F3. Interestingly, patients with F1‑F3 displayed 12.3-fold increase in *H. pylori* antigen level over patients with F0 (Fig. 2b). On the other hand, cirrhotic patients displayed a 16.52-fold and 1.34-fold increase in *H. pylori* antigen level over F0 and F1‑F3, respectively, as shown in Fig. 2c, d.

Laboratory characteristics of CHC patients with and without *H. pylori* infection are summarized in Table 1. Overall, there were 95/249 (38.15%) with no fibrosis (F0), 90/249 (36.15%) with liver fibrosis (F1‑F3), and 64/249 (25.70%) with liver cirrhosis (F4) as seen in Table 2. Next, the characteristics of patients with no fibrosis (F0), liver fibrosis (F1‑F3), and liver cirrhosis (F4) are summarized in Table 2. In this work, our patients were predominantly male (79.19%), with a mean (± SD) age of 39.63 (± 11.61) years at the time of liver biopsy. Patients with no fibrosis (F0) were young as compared to those who developed liver fibrosis (F1‑F3) or liver cirrhosis (F4) with an extremely significant difference (*P* < 0.0001) based on the analysis of one-way ANOVA followed by Tukey’s post hoc test as presented in Table 2. As anticipated, patients who developed liver cirrhosis (F4) were associated with higher levels of alanine aminotransferase (ALT), aspartate aminotransferase (AST), and alpha fetoprotein (AFP) than those who have liver fibrosis (F1‑F3) or those with no fibrosis (F0) with a significant difference (*P* < 0.05). On the other hand, the mean value of albumin and platelet count decreased with the progression of liver fibrosis being lower in patients who developed liver cirrhosis (F4) than those with no liver fibrosis (F0) and liver fibrosis (F1‑F3).

**Table 1:** Comparison between laboratory characteristics of individuals included in this work based on *Helicobacter pylori* (*H. pylori*) infection (*n* = 249)

Variables ^a^	Group I (*n* = 131)	Group II (*n* = 118)	*P* value
	Non *H. pylori* infection	*H. pylori* infection	
Sex (male/female)	97/34	82/36	0.481
ALT (U/L)	34.92 ± 2.28	48.78 ± 3.00	< 0.0001*
AST (U/L)	35.48 ± 2.50	51.97 ± 3.35	< 0.0001*
Total bilirubin (mg/dl)	1.00 ± 0.15	1.31 ± 0.10	0.087
Albumin (g/L)	4.05 ± 0.09	3.31 ± 0.07	< 0.000*
Platelets (10^9^/L)	235.87 ± 11.42	182.77 ± 10.76	0.001*
AFP (U/L)	10.79 ± 2.47	35.44 ± 10.07	0.013*

Variables were expressed as mean ± SEM

^a^Reference values: alanine aminotransferase (ALT) (male up to 41 U/L, female up to 31 U/L); aspartate aminotransferase (AST) (male up to 37 U/L, female up to 31 U/L); total bilirubin up to 1 mg/dL; albumin 3.8‑5.4 g/dL; platelet count 150‑400 × 10^9^/L; alpha fetoprotein (AFP) up to 10 U/L

*P < 0.05 is considered significant

**Table 2:** Comparison between laboratory characteristics of individuals included in this work based on METAVIR fibrosis stages (*n* = 249)

Variables^a^	Group I (*n* = 95), no fibrosis	Group II (*n* = 90), fibrosis	Group III (*n* = 64), cirrhosis	Tukey’s post hoc test		
				Group I vs. II	Group I vs. III	Group II vs. III
Age (year)	32.26 ± 0.65	43.32 ± 1.21	54.00 ± 2.08	< 0.0001*	< 0.0001*	< 0.0001*
ALT (U/L)	22.36 ± 0.92	54.07 ± 2.45	67.87 ± 3.59	< 0.0001*	< 0.0001*	< 0.0001*
AST (U/L)	24.09 ± 1.23	56.87 ± 3.39	68.97 ± 4.35	< 0.0001*	< 0.0001*	0.014*
Total bilirubin (mg/dl)	0.71 ± 0.03	1.79 ± 0.30	1.78 ± 0.18	< 0.0001*	< 0.0001*	1.00
Albumin (g/L)	3.88 ± 0.07	3.48 ± 0.17	2.97 ± 0.11	0.030*	< 0.0001*	0.030*
Platelets (10^9^/L)	240.06 ± 5.43	172.03 ± 22.24	130.67 ± 21.77	0.015*	< 0.0001*	0.259
AFP (U/L)	1.35 ± 0.06	37.54 ± 6.98	94.18 ± 23.95	0.001*	< 0.0001*	< 0.0001*

Variables were expressed as mean ± SEM

^a^Reference values: alanine aminotransferase (ALT) (male up to 41 U/L, female up to 31 U/L); aspartate aminotransferase (AST) (male up to 37 U/L, female up to 31 U/L); total bilirubin up to 1 mg/dL; albumin 3.8‑5.4 g/dL; platelet count 150‑400 × 10^9^/L; alpha fetoprotein (AFP) up to 10 U/L

*P < 0.05 is considered significant

Furthermore, the prevalence of 58-kDa *H. pylori* antigen in this category of patients was found to increase with the progression of liver disease as it was 44.45% in liver fibrosis and 71.88% in liver cirrhosis as shown in Fig. 2e. Our findings showed that *H. pylori* antigen significantly correlated with liver fibrosis stages producing a Spearman’s rank correlation coefficient (*r*) of 0.501 (*P* < 0.0001) as depicted in Fig. 2f.

Levels of different biochemical markers were determined in CHC patients with and without *H. pylori* as shown in Table 3. The results demonstrated that each METAVIR fibrosis group was accompanied by an increase in their levels in case of co-infection compared to patients at the same stage in mono-infection. On the other hand, each fibrosis group was associated with a drop in the levels of both albumin and platelet count in case of co-infection compared to HCV mono-infection but the difference was statistically not significant.

**Table 3:** Levels of laboratory parameters in different METAVIR fibrosis stages in patients with hepatitis C virus mono-infection and HCV/*Helicobacter pylori* (*H. pylori*) co-infection

Variables^a^	No fibrosis (F0)			Liver fibrosis (F1‑F3)			Cirrhosis (F4)		
	Group I^b^ (*n* = 63)	Group II^b^ (*n* = 32)	*P value	Group I^b^ (*n* = 50)	Group II^b^ (*n* = 40)	*P value	Group I^b^ (*n* = 18)	Group II^b^ (*n* = 46)	*P value
Age (year)	32.05 ± 0.91	32.50 ± 0.92	0.732	40.58 ± 1.55	46.60 ± 1.66	0.011*	55.89 ± 3.87	53.23 ± 2.50	0.570
ALT (U/L)	19.50 ± 0.90	25.75 ± 1.51	< 0.001*	49.28 ± 2.36	60.05 ± 4.80	0.027*	60.11 ± 5.64	70.91 ± 4.39	0.040*
AST (U/L)	19.42 ± 0.85	29.63 ± 2.11	< 0.001*	49.20 ± 2.94	66.45 ± 6.14	0.010*	65.17 ± 6.66	70.46 ± 5.52	0.046*
T. bilirubin (mg/dl)	0.58 ± 0.03	0.86 ± 0.04	< 0.001*	1.73 ± 0.23	1.84 ± 0.56	0.868	1.72 ± 0.44	1.81 ± 0.19	0.817
Albumin (g/L)	4.25 ± 0.07	3.45 ± 0.08	< 0.001*	3.57 ± 0.30	3.41 ± 0.21	0.666	3.23 ± 0.20	2.89 ± 0.12	0.136
Platelets (10^9^/L)	239.5 ± 7.2	240.7 ± 8.4	0.920	200.2 ± 35.1	143.9 ± 24.5	0.224	245.5 ± 121.5	110.7 ± 13.6	0.025*
AFP (U/L)	1.04 ± 0.08	1.70 ± 0.05	< 0.001*	26.38 ± 3.93	71.04 ± 19.71	0.003*	36.14 ± 4.87	106.96 ± 22.39	0.030*

Variables were expressed as mean ± SEM

^a^Reference values: alanine aminotransferase (ALT) (male up to 41 U/L, female up to 31 U/L); aspartate aminotransferase (AST) (male up to 37 U/L, female up to 31 U/L); total bilirubin (T. bilirubin) up to 1 mg/dL; albumin 3.8‑5.4 g/dL; platelet count 150‑400 ×10^9^/L; alpha fetoprotein (AFP) up to 10 U/L

^b^Group I: hepatitis C virus mono-infection; Group II: hepatitis C virus/*Helicobacter pylori* co-infection

*P < 0.05 is considered significant

Next, odds ratio was used as a measure of association between 58-kDa *H. pylori* antigen and different groups of diseases and the results are shown in Table 4. The risk for developing liver fibrosis and cirrhosis was found to increase upon *H. pylori* co-infection when compared to CHC mono-infected patients. Our findings showed that patients co-infected with *H. pylori* and HCV are 3.19 times (219%) more likely to experience cirrhosis than those who mono-infected with HCV. This may give a clue that *H. pylori* infection may have a potential role in liver-disease progression.

**Table 4:** Prevalence of *Helicobacter pylori* (*H. pylori*) antigen in different METAVIR fibrosis stages in patients with hepatitis C

Fibrosis stages	***H. pylori*** prevalence		Odds ratio, ***P***	***X***^**2**^, ***P***
	Negative	Positive		
***No fibrosis*** **(*F0*) vs.** ***fibrosis*** **(*F1‑F3*)**				
F0 (*n* = 95)	63 (66.32%)	32 (33.68%)	1.5, *P* > 0.05	2.3, *P* > 0.05
F1‑F3 (*n* = 90)	50 (55.55%)	40 (44.45%)		
***Fibrosis*** **(*F1‑F3*) vs.** ***cirrhosis*** **(*F4*)**				
F1‑F3 (*n* = 90)	50 (55.55%)	40 (44.45%)	3.19, *P* = 0.001*	11.4, *P* = 0.001*
F4 (*n* = 64)	18 (28.12%)	46 (71.88%)		
***Non cirrhosis*** **(*F0‑F3*) vs.** ***cirrhosis*** **(*F4*)**				
F0‑F3 (*n* = 185)	113 (61.08%)	72 (38.92%)	4.0, *P* < 0.0001*	20.71, *P* < 0.0001*
F4 (*n* = 64)	18 (28.12%)	46 (71.88%)		

*P < 0.05 is considered significant

## Discussion

There is some evidence to suggest that *H. pylori* infection may be associated with an increased risk of gastric and extra-gastric malignancies [2, 13–16]. Many of these studies were depending on detection of *H. pylori* antibodies in serum by ELISA [4, 17], histopathology methods [18], or on detection of bacterial deoxyribonucleic acid (DNA) in tumor tissues using polymerase chain reaction technique (PCR) [5]. Clearly, the use of antibodies-based methods can lead to ambiguous results especially since antibodies can be detectable a long time after the bacteria have ceased to colonize the gut [19]. The present work preferred to use *H. pylori* antigen-based method in order to examine the association between *H. pylori* infection and different liver diseases. In general, more efforts were performed for identifying various *H. pylori* antigens-based western blot technique, e.g., 125 kDa CagA antigen and 87 kDa VacA antigen [20]. The 58-kDa *H. pylori* antigen was identified in serum of patients with different gastric and duodenal diseases. The molecular mass of the serum antigen is similar to the 58-kDa fragment of the 87-kDa cytotoxin domain of the VacA protein, the subunit cellular antigen of the native *H. pylori* catalase, and the *H. pylori* catalase gene product [9]. In this work, *H. pylori* antigen enabled the correct identification of *H. pylori* patients showing an AUC of 0.946 with 90% sensitivity and 90% specificity. These results are almost matching with those of Attallah et al. [9] and Kabir [21] who showed comparable sensitivity (91‑98%) and specificities (83‑100%). So, using a 58-kDa *H. pylori* antigen-based method was accurate enough for studying the *H. pylori* association with different gastrointestinal cancers. On the other hand, we also aimed to investigate the 58-kDa *H. pylori* antigen in sera of patients with different liver pathology to assess the potential cofactor role of *H. pylori* infection in the progression of HCV-related chronic liver disease. In this work, 58-kDa *H. pylori* antigen was positive in 71.88% of liver cirrhosis patients compared with 44.45% in liver fibrosis patients. Other studies have evidenced of high seroprevalence of *H. pylori* antibodies among cirrhotic patients [4, 13]. Our results are very close to that of Dore et al. [13] who detected anti*-H. pylori* IgG in serum of 39% chronic hepatitis and 58% liver cirrhosis patients. In addition, Rocha et al. [5] found that *Helicobacter* DNA was high 68% in liver tissue of patients with liver cirrhosis. It was suggested that these bacteria could be implicated in the progression of liver disease to cirrhosis and hepatocellular carcinoma (HCC). In addition and consistent to our findings, Wang et al. found a strong association between *H. pylori* and chronic hepatitis C, particularly during the HCV progression stage [22]. Surprisingly, Wang et al. showed that the odds ratio for cirrhosis was 4.4 which was almost similar to that obtained in our work. Huang et al. [23] postulated that it is possible for *H. pylori* to invade the liver and gall bladder via the bile duct. Many theories have been proposed to explain the mechanism of *H. pylori* involvement in the pathogenesis and progression of cirrhosis, particularly in HCV-infected individuals [24]. Enteric *Helicobacter* species have been found to produce toxins that might cause hepatocellular damage in vivo. Many studies have tried to explain the mechanisms by increased levels of proinflammatory cytokines such as interleukins 1, 6 (IL-1, IL-6), tumor necrosis factor, and by the presence of lymphocytic mononuclear infiltrate and lymphoid follicle formation [23]. According to Abdel-Hady et al. [25], *H. pylori* infection might have a role in increasing the circulating levels of ammonia and endotoxins in cirrhotic patients, thus facilitating the onset of hepatic encephalopathy and of hepatic inflammation by stimulating the secretion of proinflammatory cytokines. As *H. pylori* infection increased hepatic AFP expression level and carbon tetrachloride (CCL_4_) intoxication increased transforming growth factor-beta1 (TGF-b1) levels, both in serum and tissues, *H. pylori* chronic infection might contribute to HCC progression from CCL4-induced fibrosis or cirrhosis [26, 27].

## Conclusion

In summary, this work showed that the elevated levels of *H. pylori-antigen* in HCV/*H. pylori* co-infection suggest increased susceptibility of co-infected patients for promoting hepatic fibrosis progression. This suggests the importance of *H. pylori* screening of patients with CHC, particularly those with HCV-related cirrhosis, in order to choose the proper treatment.

## Data Availability

Not applicable.

## Abbreviations

AFP: Alpha fetoprotein
ALP: Alkaline phosphatase
ALT: Alanine aminotransferase
AST: Aspartate aminotransferase
CCL_4_: Carbon tetrachloride
CHC: Chronic hepatitis C
DNA: Deoxyribonucleic acid
*H. pylori*: *Helicobacter pylori*
HCC: Hepatocellular carcinoma
HCV: Hepatitis C virus
IL: Interleukins
PCR: Polymerase chain reaction technique
RNA: Ribonucleic acid
TGF-b1: Transforming growth factor-beta1
